# Comparing Diagnostic Accuracy of Kato-Katz, Koga Agar Plate, Ether-Concentration, and FLOTAC for *Schistosoma mansoni* and Soil-Transmitted Helminths

**DOI:** 10.1371/journal.pntd.0000754

**Published:** 2010-07-20

**Authors:** Dominik Glinz, Kigbafori D. Silué, Stefanie Knopp, Laurent K. Lohourignon, Kouassi P. Yao, Peter Steinmann, Laura Rinaldi, Giuseppe Cringoli, Eliézer K. N'Goran, Jürg Utzinger

**Affiliations:** 1 Department of Epidemiology and Public Health, Swiss Tropical and Public Health Institute, Basel, Switzerland; 2 University of Basel, Basel, Switzerland; 3 Université de Cocody-Abidjan, Abidjan, Côte d'Ivoire; 4 Centre Suisse de Recherches Scientifiques, Abidjan, Côte d'Ivoire; 5 National Institute of Parasitic Diseases, Chinese Center for Disease Control and Prevention, Shanghai, People's Republic of China; 6 Department of Pathology and Animal Health, Faculty of Veterinary Medicine, University of Naples ‘Federico II’, Regional Center for Monitoring Parasites (CREMOPAR), Naples, Italy; Fundacao Oswaldo Cruz, Centro de Pesquisas Rene Rachou, Laboratorio de Imunologia, Brazil

## Abstract

**Background:**

Infections with schistosomes and soil-transmitted helminths exert a considerable yet underappreciated economic and public health burden on afflicted populations. Accurate diagnosis is crucial for patient management, drug efficacy evaluations, and monitoring of large-scale community-based control programs.

**Methods/Principal Findings:**

The diagnostic accuracy of four copromicroscopic techniques (i.e., Kato-Katz, Koga agar plate, ether-concentration, and FLOTAC) for the detection of *Schistosoma mansoni* and soil-transmitted helminth eggs was compared using stool samples from 112 school children in Côte d'Ivoire. Combined results of all four methods served as a diagnostic ‘gold’ standard and revealed prevalences of *S. mansoni*, hookworm, *Trichuris trichiura*, *Strongyloides stercoralis* and *Ascaris lumbricoides* of 83.0%, 55.4%, 40.2%, 33.9% and 28.6%, respectively. A single FLOTAC from stool samples preserved in sodium acetate-acetic acid-formalin for 30 or 83 days showed a higher sensitivity for *S. mansoni* diagnosis (91.4%) than the ether-concentration method on stool samples preserved for 40 days (85.0%) or triplicate Kato-Katz using fresh stool samples (77.4%). Moreover, a single FLOTAC detected hookworm, *A. lumbricoides* and *T. trichiura* infections with a higher sensitivity than any of the other methods used, but resulted in lower egg counts. The Koga agar plate method was the most accurate diagnostic assay for *S. stercoralis*.

**Conclusion/Significance:**

We have shown that the FLOTAC method holds promise for the diagnosis of *S. mansoni*. Moreover, our study confirms that FLOTAC is a sensitive technique for detection of common soil-transmitted helminths. For the diagnosis of *S. stercoralis*, the Koga agar plate method remains the method of choice.

## Author Summary

Infections with parasitic worms (e.g., *Schistosoma mansoni*, hookworm, roundworm, whipworm, and threadworm) are still widespread in the developing world. Accurate diagnosis is important for better patient management and for monitoring of deworming programs. Unfortunately, methods to detect parasite eggs or larvae in stool samples lack sensitivity, particularly when infection intensities are low. The most widely used method for the diagnosis of *S. mansoni*, hookworm, roundworm and whipworm in epidemiological surveys is the Kato-Katz technique. Recently, the FLOTAC technique has shown a higher sensitivity than the Kato-Katz method for the diagnosis of hookworm, roundworm and whipworm, but no data are available for *S. mansoni*. We compared the diagnostic accuracy of the FLOTAC with the Kato-Katz, ether-concentration and Koga agar plate techniques for *S. mansoni* and other parasitic worm infections using stool samples from 112 school children from Côte d'Ivoire. FLOTAC showed the highest sensitivity for *S. mansoni* diagnosis. Egg counts, however, were lower when using FLOTAC, an issue which needs further investigations. The FLOTAC, Kato-Katz and ether-concentration techniques failed to accurately detect threadworm larvae, and hence, the Koga agar plate remains the method of choice for this neglected parasite.

## Introduction

Schistosomiasis and soil-transmitted helminthiasis are widespread in many parts of the developing world where they negatively impact on human health and wellbeing, and thus exacerbate poverty [1]–[4]. Schistosomiasis is caused by blood flukes of the genus *Schistosoma*; in Africa both *S. mansoni* and *S. haematobium* are endemic [2], [5]–[7]. Soil-transmitted helminthiasis is caused by intestinal nematodes; hookworms (*Ancylostoma duodenale* and *Necator americanus*), the roundworm (*Ascaris lumbricoides*), the whipworm (*Trichuris trichiura*), and the threadworm (*Strongyloides stercoralis*) [1], [3], [8], [9]. A detailed understanding of the epidemiology of these parasitic worm infections is important for the design, implementation, monitoring, and evaluation of helminth control programs [10], [11].

Commonly used diagnostic methods for these parasites rely on the detection of helminth eggs or larvae in human stool. These copromicroscopic approaches have drawbacks, such as low sensitivity for the detection of light-intensity infections [12], [13]. At present, the Kato-Katz technique is the most widely used copromicroscopic method in epidemiological surveys pertaining to human intestinal helminth infections because of its simplicity [14], low cost, and the established system to stratify infection intensity into different classes based on cut-offs of egg-counts [15]. The small amount of stool analyzed (usually 41.7 mg) explains why the Kato-Katz technique has a low sensitivity to detect eggs whenever they are present at low frequency or appear highly clustered (theoretical analytic sensitivity = 24 eggs per gram of stool (EPG)) [16], [17]. The sensitivity can be increased by examining multiple Kato-Katz thick smears prepared from the same stool sample or, better yet, from multiple stool samples [12],[18]–[22]. This point highlights the appeal of parasitological diagnostic methods capable of screening a larger amount of stool, e.g., 0.5 g or even 1 g in the case of the ether-concentration method [23] or the FLOTAC technique [24].

The ether-concentration method is often used for the diagnosis of helminth infections, particularly in specialized laboratories [23], [25]–[27]. Importantly, it allows the concurrent diagnosis of intestinal protozoa, and is sometimes used in combination with the Kato-Katz method to enhance diagnostic sensitivity for helminths, and hence to deepen our understanding of polyparasitism [28]–[30]. An important feature of the ether-concentration method is that it uses preserved stool samples, fixed in either sodium acetate-acetic acid-formalin (SAF) [23], or diluted formalin [24], thus allowing sample storage and analysis at later time points. However, considerable inter-laboratory discrepancies have been noted for helminth and particularly intestinal protozoa diagnosis [26], . The Koga agar plate technique allows direct observation of *S. stercoralis* and hookworm larvae hatched from fresh stool samples incubated on nutrient agar in a humid chamber, usually after 48 hours [31].

Recent studies suggest that the FLOTAC technique [24] holds promise for the diagnosis of soil-transmitted helminth infections in humans [32]–[34]. Its potential for the diagnosis of *S. mansoni* has yet to be investigated. The FLOTAC technique takes advantage of the fact that during flotation, parasitic elements such as helminth eggs gather in the apical portion of the flotation column and can be readily translated, i.e., cut transversally for subsequent viewing under a microscope. Thus, parasitic elements are separated from fecal debris, facilitating their identification and enumeration. Protocols have been developed for the FLOTAC basic technique (theoretical analytic sensitivity = 1 EPG), the FLOTAC dual technique, the FLOTAC double technique, and the FLOTAC pellet technique (all: theoretical analytic sensitivity = 2 EPG) [24]. Importantly, in a single FLOTAC examination usually ∼1 g of stool is analyzed, and hence a single FLOTAC allows a 24-fold higher amount of stool to be examined than a single Kato-Katz thick smear, which is an important factor explaining the higher sensitivity of the FLOTAC technique.

The aim of this study was to compare the diagnostic accuracy of different techniques and sampling efforts, i.e., single and multiple Kato-Katz thick smears, ether-concentration and the FLOTAC method, for the detection and quantification of helminth eggs. The performance of the Koga agar plate technique for the detection of helminth larvae was also assessed. Particular emphasis was placed on the diagnosis of *S. mansoni*.

## Methods

### Ethical considerations and treatment

The study was cleared by the institutional research commissions of the Swiss Tropical and Public Health Institute (Basel, Switzerland) and the Centre Suisse de Recherches Scientifiques (CSRS; Abidjan, Côte d'Ivoire), and was approved by local and national health authorities of Côte d'Ivoire. The school director and teachers were informed about the objectives and procedures of the study. Parents, legal guardians, and children were informed about the study and sufficient time was given to ask questions. Written informed consent was obtained from the parents or legal guardians of all participating children. Participation was voluntary and children could withdraw from the study at any time without further obligations. At the end of the study, all children attending the school of Azaguié-IRFA (“Institut de Recherches sur les Fruits et Agrumes”) were treated free of charge with praziquantel (single 40 mg/kg oral dose using a ‘dose-pole’) and mebendazole (single 500 mg oral dose) according to WHO recommendations [35]. Additionally, children infected with *S. stercoralis* were treated with ivermectin (single 200 µg/kg oral dose).

### Study area and participants

The study was carried out in June 2008 in the primary school of Azaguié-IRFA, located in a rural setting of Azaguié in the region of Agboville, south Côte d'Ivoire (geographical coordinates: 05°36′10.5″ N latitude, 04°00′58.5″ W longitude). The village is located 56 km north of Abidjan, the economic capital of Côte d'Ivoire. In the school year 2007/2008, 200 children attended grades 1–6. We aimed for a sample size of 120 school children, similar to our preceding FLOTAC research carried out elsewhere in Côte d'Ivoire [32]. Allowing for a drop out of ∼10%, we randomly selected 133 school children from all grades and invited them to submit a single fresh morning stool.

### Field and laboratory procedures

School children were given plastic containers (125 ml) for stool collection. Upon submission, each container was labeled with a unique identification number and transferred within 1–2 hours to the CSRS in Abidjan. Stool samples were processed at the day of collection, usually within 2–3 hours after reaching the laboratory. Stool specimens were collected over six consecutive days (one sample from each of 12 children on day 1 and one sample from each of 20 children on the following 5 days).

**Figure 1:**
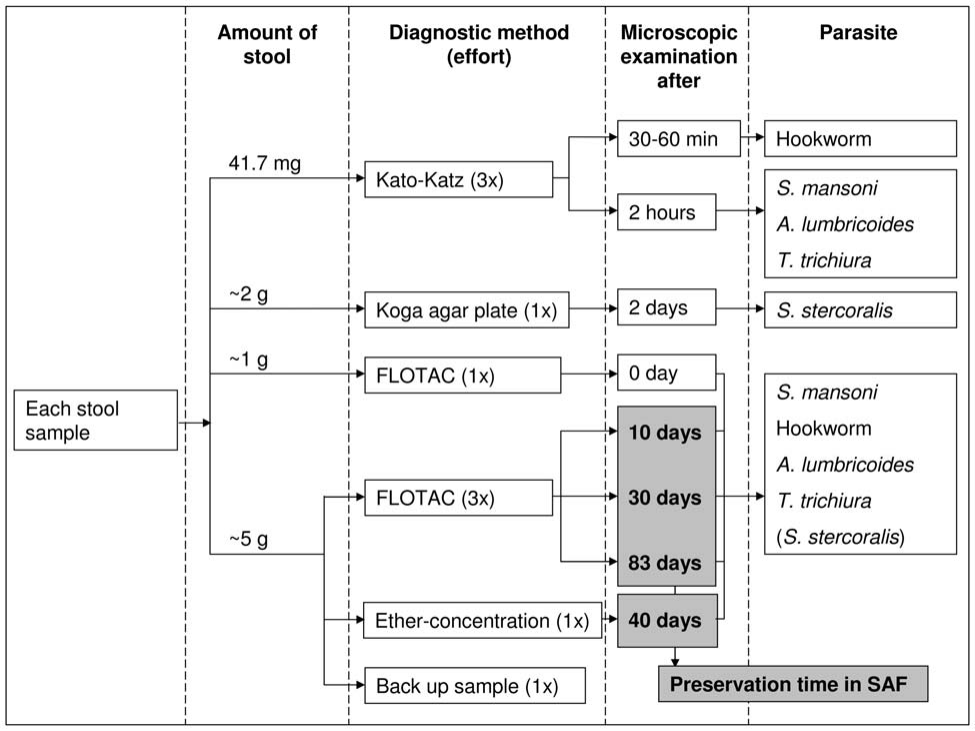
Diagnostic methods used to detect *S. mansoni* and soil-transmitted helminth infections. The flowchart details the diagnostic approaches and their temporal sequence, as well as the amount of stool examined for the detection of helminth eggs and larvae, and the comparison of the different diagnostic tools applied to 112 stool samples from school children in Azaguié-IRFA, Côte d'Ivoire, in June 2008.

As depicted in Figure 1, each fresh stool sample was subjected to the Kato-Katz method (3 thick smears), the Koga agar plate method (1 examination), and the FLOTAC dual technique (1 examination, fresh stool sample homogenized in SAF). Additionally, ∼5 g of the fresh stool were preserved in 25 ml SAF. After 9 days of preservation, these samples were filtered in order to remove large fibers (wire mesh aperture: 250 µm) and split into 5 sub-samples, each weighing ∼1 g. Among them, 3 sub-samples were examined with the FLOTAC dual technique 10, 30, and 83 days post-stool collection, 1 sub-sample was subjected to the ether-concentration method 40 days post-stool collection, and the fifth sub-sample was used as back-up and was finally discarded.

Processing of stool samples was as follows. First, triplicate Kato-Katz thick smears (41.7 mg each) were prepared. Slides were read twice; first within 30–60 min for detection of hookworm eggs and again after ∼2 hours for diagnosis of *S. mansoni*, *A. lumbricoides*, *T. trichiura*, and other helminths. For each helminth species, the number of eggs was counted under a microscope by one of four experienced laboratory technicians and recorded separately for both readings.

Second, a hazelnut-sized stool sample (average weight = 2.29 g) was placed in the middle of an agar plate. The plates were incubated in a humid chamber at 28°C for 48 hours. Plates were inspected under a microscope for the characteristic traces of hookworm and *S. stercoralis* larvae. Subsequently, the plates were rinsed with SAF, the solutions centrifuged and the sediment examined for larvae under a microscope by one experienced laboratory technician. The technician was blinded to the preceding Kato-Katz test results.

Third, the samples for FLOTAC were prepared as follows: the filtered stool suspensions were filled up to 10 ml with SAF and equally split into two 15-ml Falcon tubes. Three ml of ether were added to each tube to facilitate the removal of fatty compounds that could interfere with egg detection under a microscope. The Falcon tubes were shaken rigorously for at least 1 min and then centrifuged for 2 min at 170 g. The supernatant was discarded and each pellet suspended in 6 ml of either flotation solution FS4 (sodium nitrate: 315 g NaNO_3_ suspended in 685 ml H_2_O; specific gravity (s.g.) = 1.20) or FS7 (zinc sulphate: 685 g ZnSO_4_ H_2_O suspended in 685 ml H_2_O; s.g. = 1.35) [24]. In the present study, the FLOTAC dual technique was employed (i.e., one of two different FS in each of the two chambers of the same FLOTAC apparatus). From a panel of 14 different FS [36], we selected FS4 because it had proved to be useful for soil-transmitted helminth diagnosis in previous studies carried out in Côte d'Ivoire and Zanzibar [32], [33]. FS7 was chosen because it was particularly suitable for the detection of *S. mansoni* eggs in fecal samples obtained from infected mice and hamster (J. Keiser, L. Rinaldi, and J. Utzinger; unpublished data). Each suspension was transferred into one of the two 5-ml chambers of the FLOTAC apparatus. The apparatus was centrifuged (using a Hettich Universal 320 centrifuge; Tuttlingen, Germany) at 160 g for 5 min. Subsequently, the upper portion in the FLOTAC apparatus was translated, and the observation grid examined under a microscope at 100× magnification by one experienced laboratory technician. The technician was blinded to the preceding Kato-Katz and ether-concentration test results. Helminth eggs were counted and reported for each species in each chamber separately.

Fourth, the ether-concentration method was performed as described in detail elsewhere [27].

### Data management and statistical analysis

Data were double-entered in Microsoft Office Access 2007, cross-checked using EpiInfo (TM 3.4.1), and analyzed with STATA version 9.1 (StataCorp LP; College Station, TX). The different diagnostic methods were compared using 2-way contingency tables of frequencies, and agreement between 2 diagnostic techniques was determined (e.g., Kato-Katz *versus* ether-concentration, and Kato-Katz *versus* FLOTAC for *S. mansoni* diagnosis). Kappa (ĸ) statistic was employed to determine the strength of agreement using the following cut-offs: (i) ĸ<0.00 indicating no agreement; (ii) ĸ = 0.00–0.20 indicating poor agreement; (iii) ĸ = 0.21–0.40 indicating fair agreement; (iv) ĸ = 0.41–0.60 indicating moderate agreement; ĸ = 0.61–0.80 indicating substantial agreement; and (v) ĸ = 0.81–1.00 indicating almost perfect agreement [37], [38]. It is important to note that ĸ values depend on the marginal distributions of contingency tables. We employed a test of marginal homogeneity and, since significant values were obtained, raked ĸ values were calculated throughout [39], [40].

The sensitivity and negative predictive value (NPV) were calculated for each method, considering the combined results from all different methods, FS, and at all time points investigated, as diagnostic ‘gold’ standard. Hence, any positive test result, regardless of the technique employed, was considered a true-positive result. Following this approach specificity was set to be 100%, justified by the direct observation of parasite eggs [21], [26], [32], [33].

Helminth-specific egg counts were expressed as EPGs for three among the four techniques. For the Kato-Katz method, the number of helminth eggs in each of the three thick smears prepared from a single fresh stool sample collected from each participant were added and then multiplied by a factor 8 to obtain EPGs. For the ether-concentration method, EPGs were estimated by dividing the number of helminth eggs with the amount of stool examined after 40 days of preservation (∼1 g; see Figure 1), that is one-fifth of the total amount of weighed stool that was preserved and used for four different tests with the remaining fifth part of the preserved stool sample finally discarded. For the examination of stool samples with the FLOTAC dual technique, FS-specific EPGs were estimated as follows. For the fresh stool sample (∼1 g; see Figure 1), the number of counted helminth eggs was divided by the exact amount of stool and multiplied with a factor 2 (stool sample was equally aliquoted to one of the two FLOTAC chambers) and, additionally, with a factor 1.2 (stool sample was solved in 6 ml FS, but only 5 ml was filled into the FLOTAC apparatus). For the SAF-preserved stool, the number of counted helminth eggs was divided by one-fifth of the total amount of weighed stool (∼1 g; see Figure 1), and then multiplied by factors 2 and 1.2 as described above. Since EPGs were not normally distributed (as assessed by quantile normal plots), the geometric mean (GM), including 95% confidence intervals (CI), was calculated and graphically displayed. Our assumption was that non-overlapping 95% CIs indicate statistical significance (p<0.05).

### Post-calibration for FLOTAC

A post-calibration was performed 4 months post-stool collection to determine whether FS4 and FS7 are indeed among the most suitable FS for the FLOTAC technique for differential helminth diagnosis. For this purpose, we employed a composite human stool sample (∼100 g), pooling stool from 8 selected children with high-intensity helminth infections who provided a second stool sample just before administering anthelmintic drugs. This composite stool sample was preserved in SAF and transferred to Naples, Italy. From the panel of 14 different available FS [36], a total of 9 were selected, including FS4 and FS7. At least 3 replicates were examined for each of the 9 FS with and without a prior ether washing step, to allow an appraisal of the effect of ether on helminth diagnosis.

## Results

### Study cohort

We obtained one sufficiently large stool sample to perform triplicate Kato-Katz, multiple FLOTAC, a single ether-concentration, and a single Koga agar plate test from 112 school children. Our study cohort comprised 61 (54.5%) boys. The median age was 10 years (range: 6–15 years).

### Prevalence of *S. mansoni* and soil-transmitted helminths

**Figure 2:**
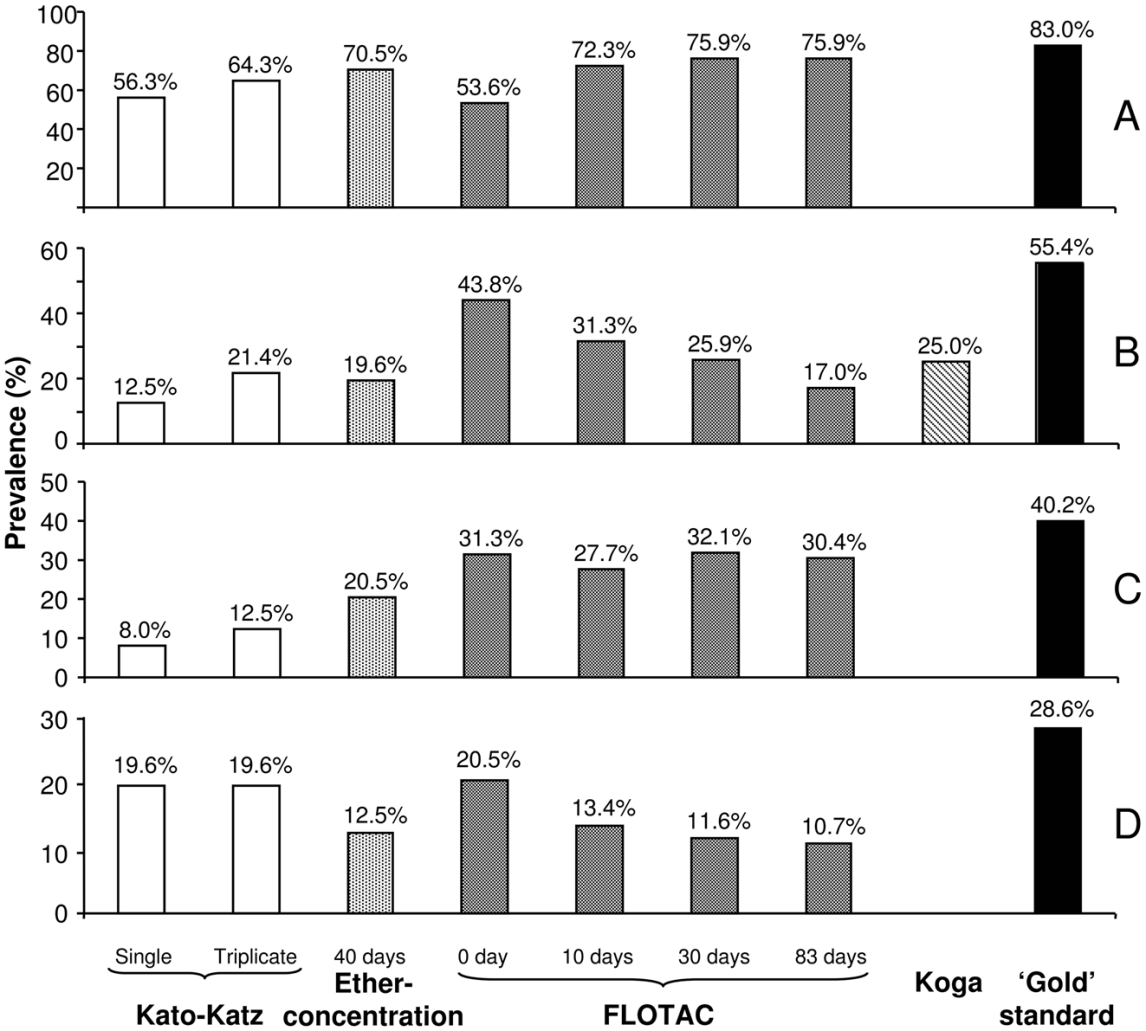
Prevalence of *S. mansoni* and soil-transmitted helminth infections. Bar charts indicate the prevalence of *S. mansoni* (A) and soil-transmitted helminth infections, i.e., hookworm (B), *T. trichiura* (C), and *A. lumbricoides* (D) among 112 school children from Azaguié-IRFA, Côte d'Ivoire, in June 2008. Results are stratified by diagnostic methods. The combined results from the different methods were considered as diagnostic ‘gold’ standard. Fresh stool examinations were subjected to triplicate Kato-Katz thick smears, a single Koga agar plate test, and a single FLOTAC examination. The fresh stool sample for FLOTAC (0 days) was homogenized in SAF. The SAF-preserved stool samples were examined once with the ether-concentration method (after 40 days) and 3 times with the FLOTAC method (at days 10, 30, and 83 post-stool collection). Prevalence estimates for *S. mansoni* using the FLOTAC method only considered the results of FS7. With regard to soil-transmitted helminth infections, the combined results of FS4 and FS7 were considered.

The combined results from the different copromicroscopic techniques were considered as diagnostic ‘gold’ standard. As shown in Figure 2, eggs of *S. mansoni* were detected in 93 children (83.0%). The overall prevalences of hookworm, *T. trichiura* and *A. lumbricoides* were 55.4%, 40.2% and 28.6%, respectively. *S. stercoralis* larvae were detected in the stools of 38 children (33.9%).

### Methods comparison for the diagnosis of *S. mansoni*

Table 1 summarizes the characteristics of 3 different techniques for *S. mansoni* diagnosis. Whilst a single Kato-Katz thick smear revealed *S. mansoni* at a prevalence of 56.3%, the cumulative prevalence after examination of 3 Kato-Katz thick smears was 64.3%; an increase of 14.2%. The observed *S. mansoni* prevalence based on a single ether-concentration test was 70.5%. Subjecting a fresh stool sample homogenized in SAF to the FLOTAC dual technique, but considering only results from FS7, revealed a *S. mansoni* prevalence of 53.6%. Preservation of stool samples in SAF for 10, 30, and 83 days, and examination with FLOTAC FS7 revealed point prevalence estimates of 72.3–75.9%.

**Table 1:** Prevalence, sensitivity, and negative predicted value (NPV) derived by different diagnostic methods.

Parasite	Technique	Number of infected school children (%)	Sensitivity in % (95% CI)	NPV (95% CI)
*S. mansoni*	‘Gold’ standard	93 (83.0)	100	100
	Kato-Katz (single)	63 (56.3)	67.7 (59.1–76.4)	38.8 (29.8–47.8)
	Kato-Katz (triplicate)	72 (64.3)	77.4 (69.7–85.2)	47.5 (38.3–56.7)
	Ether-concentration	79 (70.5)	85.0 (78.3–91.6)	57.6 (48.4–66.7)
	FLOTAC (fresh)	60 (53.6)	64.5 (55.7–73.4)	36.5 (27.6–45.5)
	FLOTAC (10 days)	81 (72.3)	87.1 (80.9–93.3)	61.3 (52.3–70.3)
	FLOTAC (30 days)	85 (75.9)	91.4 (86.2–96.6)	70.4 (61.9–78.8)
	FLOTAC (83 days)	85 (75.9)	91.4 (86.2–96.6)	70.4 (61.9–78.8)
Hookworm	‘Gold’ standard	62 (55.4)	100	100
	Kato-Katz (single)	14 (12.5)	22.6 (14.8–30.3)	51.0 (41.8–60.3)
	Kato-Katz (triplicate)	24 (21.4)	38.7 (29.7–47.7)	56.8 (47.6–66.0)
	Ether-concentration	22 (19.6)	35.5 (26.6–44.3)	55.6 (46.4–64.8)
	Koga agar plate	28 (25.0)	45.2 (35.9–54.4)	59.5 (50.4–68.6)
	FLOTAC (fresh)	49 (43.8)	79.0 (71.5–86.6)	79.4 (71.9–86.9)
	FLOTAC (10 days)	35 (31.3)	56.5 (47.3–65.6)	64.9 (56.1–73.8)
	FLOTAC (30 days)	29 (25.9)	46.8 (37.5–56.0)	60.2 (51.2–69.3)
	FLOTAC (83 days)	19 (17.0)	30.6 (22.1–39.2)	53.8 (44.5–63.0)
*T. trichiura*	‘Gold’ standard	45 (40.2)	100	100
	Kato-Katz (single)	9 (8.0)	20.0 (12.6–27.4)	65.0 (56.2–73.9)
	Kato-Katz (triplicate)	14 (12.5)	31.1 (22.5–39.7)	68.4 (59.8–77.0)
	Ether-concentration	23 (20.5)	51.1 (41.9–60.4)	75.3 (67.3–83.3)
	FLOTAC (fresh)	35 (31.3)	77.8 (70.1–85.5)	87.0 (80.8–93.2)
	FLOTAC (10 days)	31 (27.7)	68.9 (60.3–77.5)	82.7 (75.7–89.7)
	FLOTAC (30 days)	36 (32.1)	80.0 (72.6–87.4)	88.2 (82.2–94.1)
	FLOTAC (83 days)	34 (30.4)	75.6 (67.6–83.5)	85.9 (79.5–92.3)
*A. lumbricoides*	‘Gold’ standard	32 (28.6)	100	100
	Kato-Katz (single)	22 (19.6)	68.8 (60.2–77.3)	88.9 (83.1–94.7)
	Kato-Katz (triplicate)	22 (19.6)	68.8 (60.2–77.3)	88.9 (83.1–94.7)
	Ether-concentration	14 (12.5)	43.8 (34.6–52.9)	81.6 (74.5–88.8)
	FLOTAC (fresh)	23 (20.5)	71.9 (63.5–80.2)	89.9 (84.3–95.5)
	FLOTAC (10 days)	15 (13.4)	46.9 (37.6–56.1)	82.5 (75.4–89.5)
	FLOTAC (30 days)	13 (11.6)	40.6 (31.5–49.7)	80.8 (73.5–88.1)
	FLOTAC (83 days)	12 (10.7)	37.5 (28.5–46.5)	80.0 (72.6–87.4)

Number of school children (total: 112 individuals from Azaguié-IRFA, Côte d'Ivoire) diagnosed with *S. mansoni*, hookworm, *T. trichiura*, and *A. lumbricoides* in a single stool sample using different methods (Kato-Katz, ether-concentration, FLOTAC, and Koga agar plate, where appropriate), and sensitivity, and NPV of the respective technique. Fresh stool samples for FLOTAC were homogenized in SAF. Stool samples for ether-concentration and FLOTAC at day 10, 30, and 83 were preserved in SAF.

The highest sensitivity for *S. mansoni* diagnosis (91.4%) was found for the FLOTAC dual technique after 30 and 83 days of preservation in SAF. The sensitivity of a single ether-concentration test was 85.0%, whereas triplicate or only a single Kato-Katz revealed sensitivities of 77.4% and 67.7%, respectively.

Table 2 shows 2-way contingency tables comparing the results of a single FLOTAC (fresh stool, SAF preservation for 10, 30, or 83 days) and the single ether-concentration test (SAF preservation for 40 days) with the combined results of the triplicate Kato-Katz thick smear readings. Using fresh stool samples for FLOTAC (homogenized in SAF) and Kato-Katz, 51 *S. mansoni* infections were concurrently detected by both techniques, whereas 21 additional infections were diagnosed by triplicate Kato-Katz only, and 9 additional infections were detected by FLOTAC only. The agreement between these 2 methods was moderate (raked ĸ = 0.49). Comparing the results of FLOTAC performed with stool samples preserved in SAF for 10, 30, or 83 days with triplicate Kato-Katz from fresh stool, both methods concurrently detected between 69 and 71 *S. mansoni* infections, whereas 12–16 additional infections were only found by the FLOTAC technique and 1–3 infections were only detected by triplicate Kato-Katz thick smears. Substantial agreement was found for the stool samples preserved in SAF for 10 days (raked ĸ = 0.76) or 83 days (raked ĸ = 0.71), and an almost perfect agreement after 30 days of SAF preservation (raked ĸ = 0.84).

**Table 2:** Agreement between different diagnostic techniques for the detection of *S. mansoni*.

		Triplicate Kato-Katz			Raked kappa	Marginal homogeneity
		Positive	Negative	Total		P-values
FLOTAC						
Fresh stool homogenized in SAF						
	Positive	51	9	60		
	Negative	21	31	52		
	Total	72	40	112	0.49	0.029
Preserved stool in SAF (10 days)						
	Positive	69	12	81		
	Negative	3	28	31		
	Total	72	40	112	0.76	0.020
Preserved stool in SAF (30 days)						
	Positive	71	14	85		
	Negative	1	26	27		
	Total	72	40	112	0.84	<0.001
Preserved stool in SAF (83 days)						
	Positive	69	16	85		
	Negative	3	24	27		
	Total	72	40	112	0.71	0.003
Ether-concentration method						
Preserved stool in SAF (40 days)						
	Positive	69	10	79		
	Negative	3	30	33		
	Total	72	40	112	0.79	0.052

SAF: sodium acetate-acetic acid-formalin.

The 2-way contingency tables show the agreement between triplicate Kato-Katz thick smears, FLOTAC, and the ether-concentration for the diagnosis of *S. mansoni* in stool samples from 112 school children from Azaguié-IRFA, Côte d'Ivoire, in June 2008.

The ether-concentration method and triplicate Kato-Katz diagnosed 69 *S. mansoni* infections concurrently, whereas 10 infections were only found by the ether-concentration method and 3 were detected by triplicate Kato-Katz thick smears only, owing to a substantial agreement (raked ĸ = 0.79).

**Figure 3:**
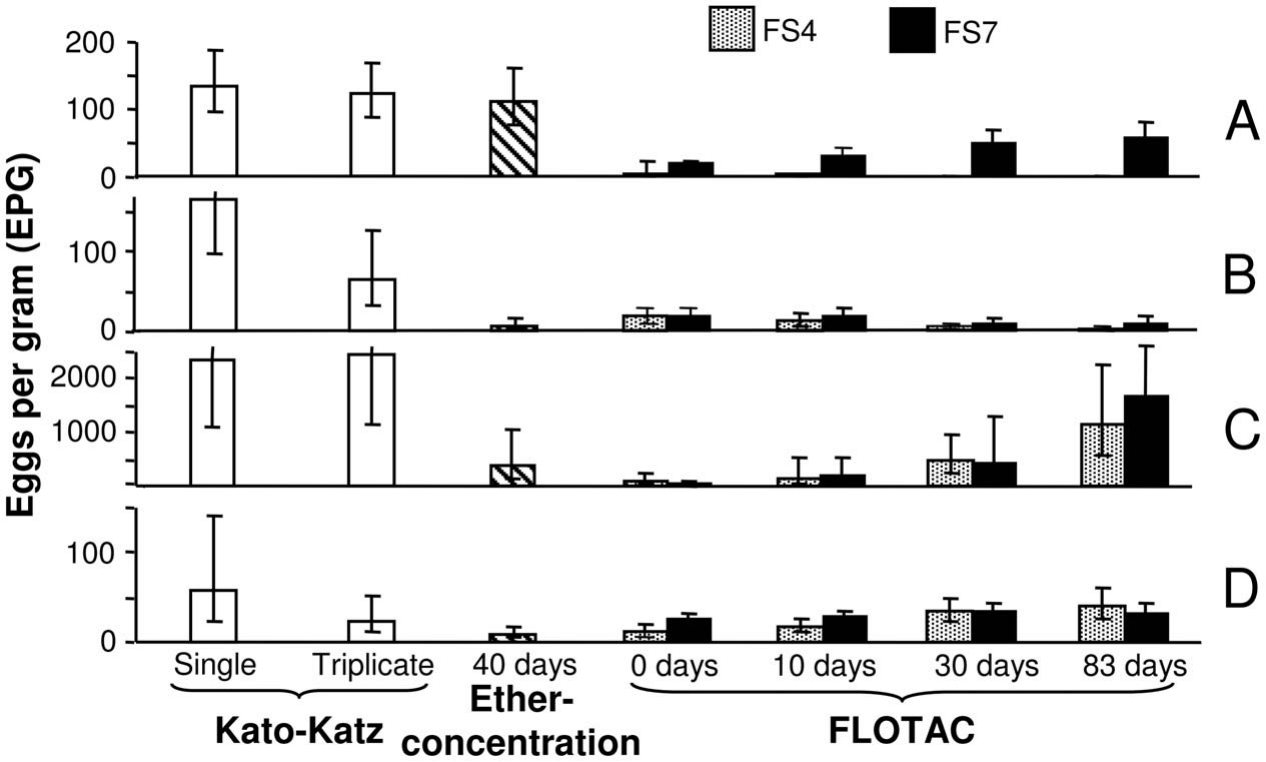
Geometric mean (GM) fecal egg counts according to different diagnostic techniques. Bar charts indicate the GM of fecal egg counts (as expressed in eggs per gram of stool (EPG) according to different techniques for the diagnosis of *S. mansoni* (A), hookworm (B), *A. lumbricoides* (C), and *T. trichiura* (D) in stool samples from 112 school children from Azaguié-IRFA, Côte d'Ivoire, in June 2008. The results for the FLOTAC method are presented separately for FS4 and FS7. Error bars indicate 95% confidence intervals (CIs) of the GM.

Triplicate Kato-Katz thick smears revealed a mean infection intensity of 121.2 EPG (95% CI: 86.8–169.2 EPG). The mean infection intensity based on a single ether-concentration examination was 110.7 EPG (95% CI: 76.0–161.1 EPG). These mean egg counts were significantly higher than those obtained with the FLOTAC dual technique, regardless of whether FS4 or FS7 were employed (Figure 3A). There was one exception: a single FLOTAC performed on stool samples preserved in SAF for 83 days and using FS7 revealed similar EPGs as triplicate Kato-Katz thick smears and a single ether-concentration test.

Using FS4 with stool preserved in SAF for 10 days resulted in the detection of only 10 (8.9%), and after 83 days of preservation of no *S. mansoni*-positive individuals. The use of FS7, on the other hand, revealed 81 (72.3%) and 85 (75.9%) infections after 10 and 83 days of preservation, respectively. While the observed *S. mansoni* fecal egg counts in FS4 decreased over time to zero, an increase was observed in FS7 from a mean of 32.3 EPG (95% CI: 24.7–42.3 EPG) at day 10 to 57.7 EPG (95% CI: 41.5–80.2 EPG) at day 83.

**Figure 4:**
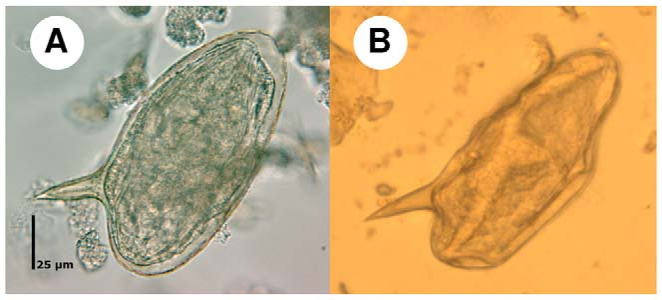
*S. mansoni* eggs detected by the Kato-Katz or FLOTAC method. The pictures show a *S. mansoni* egg as seen under a light microscope using 100× magnification. *S. mansoni* egg without deformation as seen in a Kato-Katz thick smear (A), and egg deformed through the influence of zinc sulphate in FS7 and centrifugation as seen under the FLOTAC reading disc (B).

Figure 4 shows that the shape of *S. mansoni* eggs was somewhat altered when using the FLOTAC dual technique and employing FS7. However, the characteristic lateral spine remained, and hence unambiguous diagnosis was ascertained.

### Methods comparison for the diagnosis of soil-transmitted helminths

For hookworm, the observed prevalence increased from 12.5% after a single to 21.4% after triplicate Kato-Katz examination, an increase of 71.2%. For *T. trichiura* there was an increase from 8.0% to 12.5% (+56.3%). No increase was observed for *A. lumbricoides*; a prevalence of 19.6% was obtained already after the first Kato-Katz thick smear reading (Figure 2B–D and Table 1).

Analyses with the Koga agar plate method revealed 28 (25.0%) hookworm infections. The highest observed prevalence of hookworm infection was obtained with the FLOTAC dual technique using fresh stool samples homogenized in SAF (43.8%). The observed prevalence at days 10 and 83 post-conservation decreased to 31.3% and 17.0%, respectively. A single Kato-Katz revealed a hookworm infection intensity of 165.7 EPG (95% CI: 97.0–282.4 EPG), whereas triplicate Kato-Katz thick smears suggested an intensity of less than half of this value (64.6 EPG; 95% CI: 32.8–127.2 EPG). The difference in fecal egg counts determined by the Kato-Katz and the FLOTAC techniques in both FS (fresh stool: FS4 = 18.1 EPG; 95% CI: 11.3–29.2 EPG, and FS7 = 18.7 EPG; 95% CI: 11.3–30.9 EPG) was significant. In the preserved stool samples, the observed EPGs for hookworm decreased significantly in FS4 from day 10 (13.3 EPG; 95% CI: 7.4–23.8 EPG) to day 83 (2.7 EPG; 95% CI: 1.2–5.9 EPG) (Figure 3B). In FS7, the fecal egg counts also decreased, but the difference showed no statistical significance after 10 and 83 days of SAF conservation (from 18.1 EPG (95% CI: 11.5–28.6 EPG) to 11.1 EPG (95% CI: 6.1–20.2 EPG)).

The highest prevalence of *A. lumbricoides* was estimated by a single FLOTAC from fresh stool homogenized in SAF (20.5%), but triplicate Kato-Katz showed only a marginally lower prevalence (19.6%). The examination of stool samples preserved in SAF for 83 days using FLOTAC or an ether-concentration test at day 40 after stool collection resulted in observed prevalences of 10.7% and 12.5%, respectively. The *A. lumbricoides* fecal egg counts determined with Kato-Katz were significantly higher than those obtained with FLOTAC, except for the results generated after 83 days of stool preservation. There was a significant increase for *A. lumbricoides* egg counts both with FS4 (from 148.4 EPG (95% CI: 39.9–551.5 EPG) to 1159.3 EPG (95% CI: 596.6–2252.7 EPG); a 7.8-fold increase) and with FS7 (from 181.2 EPG (95% CI: 60.0–546.9 EPG) to 1688.5 EPG (95% CI: 889.4–3205.8 EPG); a 9.3–fold increase) when comparing the 10 and 83 post-stool collection preservation time points (Figure 3C).

The highest observed *T. trichiura* prevalence (32.1%) was obtained with a single FLOTAC examined after 30 days of stool preservation in SAF. Considerably lower prevalences were obtained with a single ether-concentration test (20.5%) and triplicate Kato-Katz thick smears (12.5%). The *T. trichiura* fecal egg count for a single Kato-Katz thick smear was 56.3 EPG (95% CI: 22.4–141.4 EPG). For triplicate Kato-Katz, the respective egg count was 24.0 EPG (95% CI: 11.4–50.6 EPG). Consistently more *T. trichiura* eggs were found in FS4 than in FS7, but the difference was only significant at day 30 post-preservation (FS4 = 35.1 EPG, 95% CI: 24.4–50.4 EPG; FS7 = 15.8 EPG, 95% CI: 10.2–24.4 EPG). There was an apparent increase of egg counts over the course of stool preservation in SAF, i.e., in FS4 from 17.5 EPG (95% CI: 11.2–27.2 EPG) to 39.6 EPG (95% CI: 25.8–60.8 EPG), a 2.3-fold increase, and in FS7 from 11.8 EPG (95% CI: 7.9–17.5 EPG) to 22.2 EPG (95% CI: 14.3–34.7 EPG), a 1.9-fold increase (Figure 3D).

*S. stercoralis* larvae were found on Koga agar plates prepared with stool samples from 38 children but only 1 of these 38 infection was diagnosed after triplicate Kato-Katz examinations and 2 of these 38 infections were detected with FLOTAC in the fresh stool samples processed in SAF. No *S. stercoralis* larvae were found in the preserved samples, neither by FLOTAC nor by ether-concentration.

### Post-calibration of FLOTAC

The use of FS7 resulted in the highest *S. mansoni* fecal egg count (average: 38.3 EPG, SD: 2.7 EPG; 6 replications), but stool samples had to be washed with ether as otherwise, due to the darkening effect of organic debris, accurate reading was not feasible. For hookworm diagnosis, FS4 produced the highest fecal egg count (average: 103.2 EPG, SD: 23.6 EPG; 6 replications). Of note, hookworm eggs were readily detected only in the absence of ether for sample preparation. With regard to *A. lumbricoides* and *T. trichiura*, the results from the post-calibration were less clear-cut than those for *S. mansoni* and hookworm, as all tested FS resulted in relatively high egg count averages for *A. lumbricoides* (306.0–515.0 EPG, SD: 22.2–176.8 EPG; 3–4 replications) and *T. trichiura* (6.0–26.0 EPG, SD: 2.8–15.0 EPG; 3–4 replications), whenever an ether washing step was included.

## Discussion

Accurate diagnosis is key for adequate patient management and for guiding the design, implementation, and monitoring of community-based infectious disease control programs [13], [41]. We compared the diagnostic accuracy of two widely used techniques for detection and quantification of helminth eggs in fecal samples – the Kato-Katz thick smear using fresh stool, and the ether-concentration method using SAF-preserved samples – with the recently developed FLOTAC technique. Particular emphasis was placed on the diagnosis of *S. mansoni* because of the public health importance of intestinal schistosomiasis [2], [5]–[7], and because the FLOTAC method had not previously been investigated for this parasite. Additionally, the Koga agar plate method was employed, mainly for the diagnosis of *S. stercoralis*, but also for the detection of hookworm larvae.

Best results, i.e., high sensitivities (87.1–91.4%) for detecting *S. mansoni* eggs (prevalence: 72.3–75.9%), were achieved after stool samples were homogenized, preserved in SAF, and examined after 10–83 days with the FLOTAC dual technique. Almost as sensitive was a single ether-concentration (85.0%), revealing a *S. mansoni* prevalence of 70.5%. Triplicate Kato-Katz examinations resulted in a prevalence estimate of 64.3%. A single Kato-Katz and FLOTAC using fresh stool revealed considerably lower *S. mansoni* point prevalences of 56.3% and 53.6%, respectively. It should be noted, however that neither FLOTAC, nor the ether-concentration test, nor multiple Kato-Katz readings detected ‘all’ *S. mansoni* or ‘all’ soil-transmitted helminth infections. There was moderate to almost perfect agreement between the FLOTAC or ether-concentration method and triplicate Kato-Katz thick smears according to raked ĸ values.

With regard to soil-transmitted helminth diagnosis, our results confirm that a single FLOTAC is more sensitive than multiple Kato-Katz thick smears for the detection of hookworm, *A. lumbricoides*, and *T. trichiura* eggs in fecal samples [32]–[34]. A single FLOTAC using fresh stool processed with SAF was also more sensitive than a single ether-concentration test for the diagnosis of hookworm, *A. lumbricoides*, and *T. trichiura* infections. Finally, for hookworm diagnosis, a single FLOTAC using fresh stool was more sensitive than a single Koga agar plate test.

The design of our study allowed investigating the effect of the duration of stool preservation on helminth species-specific diagnosis. The duration of stool fixation had a considerable effect on the diagnostic performance of copromicroscopic techniques. While the number of *S. mansoni* infections detected after 10, 30, and 83 days of stool preservation in SAF remained constant (81–85 infections), there was an apparent increase in fecal egg counts from day 10 to day 83, from 32.3 EPG to 57.7 EPG (considering only FS7). The observed prevalence of hookworm and *A. lumbricoides* decreased with increasing duration of SAF conservation before FLOTAC analysis. For example, while the point prevalence of hookworm was 43.8% for FLOTAC using fresh stool, it decreased to 17.0% after stool samples had been preserved in SAF for 83 days. On the other hand, there was no apparent decline in the prevalence of *T. trichiura* over the 83-day SAF preservation period. Higher fecal egg counts were observed for *A. lumbricoides* and *T. trichiura* as a function of stool preservation duration. Regarding hookworm diagnosis, there was a sharp decrease in fecal egg counts as a function of preservation time using SAF, suggesting a negative impact of this preservation medium on hookworm eggs. However, during the post-calibration investigation and subsequent studies, it was found that the introduction of an ether washing step resulted in lower hookworm egg counts. Indeed, considerably higher hookworm egg counts were revealed in SAF-preserved stool samples in the absence of ether, whereas destroyed hookworm eggs could be observed after exposure of the sample to ether.

These observations indicate that the prolonged preservation of stool in SAF in combination with ether used for sample preparation might destroy the fragile hookworm eggs. To test this hypothesis, it will be interesting to investigate the exact influence of the preservation media alone, i.e., we still lack data on the influence of SAF and/or ether on helminth egg counts. The underlying mechanisms resulting in increasing *S. mansoni, A. lumbricoides*, and *T. trichiura* fecal egg counts estimates with time of preservation, and for the higher diagnostic sensitivity, yet lower egg counts when using FLOTAC as opposed to the Kato-Katz thick smear method, remain elusive and are the subject of ongoing deliberations and studies. We speculate that the helminth larvae are able to further develop and gain weight in these environmentally resistant eggs. This might lead to a change in density, and hence altered floating behavior of the eggs. However, these apparent fluctuations give rise to fears regarding the consistency of the diagnostic performance of FLOTAC when performed after non-standardized preservation time, on different populations, and by different laboratories.

The somewhat higher fecal egg counts of *S. mansoni*, *A. lumbricoides*, and *T. trichiura* using FLOTAC at later time points of stool conservation, and the consistently higher fecal egg counts using Kato-Katz might be explained by the following additional reasons. First, helminth eggs should not be considered “inert elements”. Instead, interactions occur between the different compartments within a floating fecal suspension (e.g., FS components, parasitic elements, fixative, ether, and residues of the host alimentation), and these might be complex [24]. New research is therefore needed to elucidate potential interactions between these compartments. Second, the high fecal egg counts derived from the Kato-Katz thick smear readings shown in Fig. 3 might be misleading. EPG values obtained from Kato-Katz thick smear readings are not continuous due to the multiplication factor used (i.e., a factor 24 for a single, and a factor 8 for triplicate Kato-Katz thick smear readings). Hence, the minimum positive value for a single measurement is 24 EPG. Third, there are additional reasons why the Kato-Katz technique might overestimate fecal egg counts. For example, when scraping the plastic spatula of the Kato-Katz kit across the upper surface of the fine-meshed screen placed on top of the stool sample, the feces is sieved, and helminth eggs are concentrated [42], [43], an issue we are currently investigating.

The available results pose considerable challenges for articulating recommendations regarding the optimal deployment of diagnostic tools for patient diagnosis, drug efficacy evaluations, and surveillance in areas where soil-transmitted helminths and *S. mansoni* are co-endemic. While the results pertaining to *S. mansoni* clearly argue for SAF-conservation of stool samples and their analysis with FS7 at a later time point, the data regarding common soil-transmitted helminth infections suggest that immediate diagnosis with FS4 should be pursued. Important trade-offs between time and fecal egg counts also exist for different soil-transmitted helminth species. Sound conclusions can probably only be drawn once more results and experience from the field are available, but it is clear that a method which needs different times and solutions to reliably diagnose distinct helminth species infections in a poly-parasitized patient is not ideal.

The current study is part of a broad attempt at validating the FLOTAC technique for human helminth diagnosis. Although we knew from previous investigations that FS4 is particularly suitable for the detection of soil-transmitted helminth eggs [32], , and prior investigations with fecal pellets obtained from *S. mansoni*-infected mice and hamster revealed that FS7 is suitable for detection of *S. mansoni* eggs, this issue has never been addressed in a systematic manner, using a single pool of stool of uniform characteristics. In a post-calibration approach, we employed a composite human stool sample of ∼100 g, pooling stool from a few selected children with high-intensity helminth infections. The results of the post-calibration underscore that the ether washing step potentially destroys hookworm eggs after a certain conservation period in SAF. No *S. mansoni* eggs were found when using FS4, and only few hookworm eggs were found in FS7. With regard to *A. lumbricoides* and *T. trichiura*, the results from the post-calibration were less clear-cut than those for *S. mansoni* and hookworm, as a number of FS resulted in high fecal egg counts for *A. lumbricoides* and *T. trichiura*.

Regarding parasitological *S. stercoralis* diagnosis, it is conventionally either done with the Koga agar plate method – as in the present study – or the Baermann method [12], [21], [31], [44]–[46]. Our study offered an opportunity to also obtain preliminary results with FLOTAC for *S. stercoralis* diagnosis. Only 2 individuals were found positive for *S. stercoralis* when fresh stool samples were subjected to FLOTAC, whereas the Koga agar plate method revealed 38 infections. One of the 2 samples determined as *S. stercoralis*-positive using the FLOTAC method contained so many larvae that they were even observed in the Kato-Katz thick smears. Of note, the tegument of the *S. stercoralis* larvae detected by FLOTAC showed signs of degeneration, which might be due to the SAF preservation, the prior washing step with ether, the FS, or a combination of these chemicals. More research is needed to determine whether the FLOTAC technique might be further adapted to allow *S. stercoralis* diagnosis.

In conclusion, the high sensitivity of a single FLOTAC examination for diagnosing common soil-transmitted helminth infections has been confirmed, but fecal egg counts are consistently lower when compared to the Kato-Katz method. This is an important issue and warrants additional studies. Importantly, we have shown that the FLOTAC method holds promise for the detection of *S. mansoni* eggs, particularly in well-homogenized stool samples after preservation in SAF for at least 10 days. Further validation of the FLOTAC technique is under way in different parts of the world, as this technique might become an indispensable tool for patient management and rigorous monitoring of anthelmintic drug efficacy studies and community-based helminth control programs.

## Supporting Information

Checklist S1 STARD checklist
(0.89 MB PDF)
