# Supplementary material for: Comparing Diagnostic Accuracy of Kato-Katz, Koga Agar Plate, Ether-Concentration, and FLOTAC for Schistosoma mansoni and Soil-Transmitted Helminths
Source: PLoS Negl Trop Dis. 2010 Jul 20;4(7):e754. doi: 10.1371/journal.pntd.0000754 (PMC2907416; doi:10.1371/journal.pntd.0000754)
Supplement: Checklist S1 — STARD checklist (0.89 MB PDF) [file pntd.0000754.s001.pdf]

Table 1. STARD checklist for the reporting of studies of diagnostic accuracy.

Section and Topic	Item #		On page #	On line #
TITLE/ABSTRACT/ KEYWORDS	1	Identify the article as a study of diagnostic accuracy (recommend MeSH heading 'sensitivity and specificity').	1+2+3	1+2, 20, 23, 43, 49
INTRODUCTION	2	State the research questions or study aims, such as estimating diagnostic accuracy or comparing accuracy between tests or across participant groups.	6	104 - 108
METHODS		Describe		
Participants	3	The study population: The inclusion and exclusion criteria, setting and locations where the data were collected.	6,7	127 - 134
	4	Participant recruitment: Was recruitment based on presenting symptoms, results from previous tests, or the fact that the participants had received the index tests or the reference standard?	7	132 - 134
	5	Participant sampling: Was the study population a consecutive series of participants defined by the selection criteria in items 3 and 4? If not, specify how participants were further selected.	7	132 - 134
	6	Data collection: Was data collection planned before the index test and reference standard were performed (prospective study) or after (retrospective study)?	9	201 - 203
Test methods	7	The reference standard and its rationale.	9, 10	202 - 205
	8	Technical specifications of material and methods involved including how and when measurements were taken, and/or cite references for index tests and reference standard.	Figure 1<br>4, 5, 7-9	70, 79, 90<br>137 - 185
	9	Definition of and rationale for the units, cutoffs and/or categories of the results of the index tests and the reference standard.	9,10	193 - 222
	10	The number, training and expertise of the persons executing and reading the index tests and the reference standard.	8,9	156-157, 162<br>182
	11	Whether or not the readers of the index tests and reference standard were blind (masked) to the results of the other test and describe any other clinical information available to the readers.	8,9	163, 182
Statistical methods	12	Methods for calculating or comparing measures of diagnostic accuracy, and the statistical methods used to quantify uncertainty (e.g. 95% confidence intervals).	10	219 - 222
	13	Methods for calculating test reproducibility, if done.	/	
RESULTS		Report		
Participants	14	When study was done, including beginning and ending dates of recruitment.	6,7	127, 139-142
	15	Clinical and demographic characteristics of the study population (e.g. age, sex, spectrum of presenting symptoms, comorbidity, current treatments, recruitment centers).	11	236-240, 243-24[illegible]
	16	The number of participants satisfying the criteria for inclusion that did or did not undergo the index tests and/or the reference standard; describe why participants failed to receive either test (a flow diagram is strongly recommended).	11	236-240
Test results	17	Time interval from the index tests to the reference standard, and any treatment administered between.	Figure 1<br>7	137 - 151
	18	Distribution of severity of disease (define criteria) in those with the target condition; other diagnoses in participants without the target condition.	/	
	19	A cross tabulation of the results of the index tests (including indeterminate and missing results) by the results of the reference standard; for continuous results, the distribution of the test results by the results of the reference standard.	Tables 1+2<br>11 - 15	249 - 344
	20	Any adverse events from performing the index tests or the reference standard.	/	
Estimates	21	Estimates of diagnostic accuracy and measures of statistical uncertainty (e.g. 95% confidence intervals).	Table 1	
	22	How indeterminate results, missing responses and outliers of the index tests were handled.	/	
	23	Estimates of variability of diagnostic accuracy between subgroups of participants, readers or centers, if done.	Figure 2+3	
	24	Estimates of test reproducibility, if done.	15, 16	346 - 355
DISCUSSION	25	Discuss the clinical applicability of the study findings.	16 - 21	357 - 477
